# Reversible occlusion of the pulmonary vasculature by transarterial embolisation with degradable starch microspheres: preclinical assessment in a human isolated lung perfusion model

**DOI:** 10.1186/s41747-021-00255-9

**Published:** 2022-02-04

**Authors:** Benedikt M. Schaarschmidt, Alexis Slama, Stéphane Collaud, Özlem Okumus, Hannah Steinberg, Sebastian Bauer, Hans-Ulrich Schildhaus, Jens Theysohn, Clemens Aigner

**Affiliations:** 1 Department of Diagnostic and Interventional Radiology and Neuroradiology, University Hospital Essen, Hufelandstraße 55, 45147 Essen, Germany; 2 Department of Thoracic Surgery and Thoracic Endoscopy, University Medicine Essen-Ruhrlandklinik, Essen, Germany; 3 Sarcoma Center, West German Cancer Center, University Hospital Essen, University of Duisburg-Essen, Essen, Germany; 4 Institute of Pathology, University Hospital Essen, University of Duisburg-Essen, Essen, Germany; 5 German Cancer Consortium (DKTK), Center Essen, Essen, Germany

**Keywords:** alpha-Amylases, Degradable starch microspheres, Lung neoplasms, Perfusion imaging, Tomography (x-ray computed)

## Abstract

**Background:**

Transpulmonary embolisation (TPE) using degradable starch microspheres (DSM) is a potential approach to treat pulmonary metastases. However, there is a paucity of detailed information on perfusion dynamics. The aim of this study was to establish a human *ex vivo* isolated lung perfusion (ILP) model to observe and evaluate the effects of DSM-TPE in a near-physiologic setting.

**Methods:**

ILP was carried out on six surgically resected lung lobes. At baseline, computed tomography (CT), including CT perfusion imaging (CTPI), and histopathological sampling were performed (t30). DSM-TPE was initiated and increased stepwise (t45, t60, t75, and t90) to be followed by CT imaging, histopathological sampling, and pulmonary arterial pressure (PAP). After the last assessment (t90), alpha-amylase was injected into the pulmonary artery to allow for DSM hydrolysation and two additional assessments (t105; t120). Histopathological specimens were evaluated using a semiquantitative ordinal score. CTPI was used for time to peak (TTP) analysis.

**Results:**

After DSM administration, PAP and TTP increased significantly: PAP slope 95% confidence interval (CI) 0.104−0.483, *p* = 0.004; TTP t30 *versus* t45, *p* = 0.046. After the addition of alpha-amylase, functional parameters reverted to values comparable to baseline. In histopathological samples, embolisation grades increased significantly until t90 (slope 95% CI 0.027−0.066, *p* < 0.001) and decreased after addition of alpha-amylase (slope 95% CI -0.060−0.012, *p* = 0.165),

**Conclusions:**

The ILP model demonstrated successfully both the physiologic effect of DSM-TPE on human lungs and its reversibility with alpha-amylase. Thus, it can be used as a near-physiologic preclinical tool to simulate and assess later clinical approaches.

## Key points

- A human *ex vivo* isolated lung perfusion model was established for investigation.
- In this near-physiological model, transpulmonary embolisation using degradable starch microspheres could be simulated and reversed successfully.
- Lung metastases could potentially be treated with transpulmonary chemoembolisation using degradable starch microspheres.

## Background

Pulmonary metastases are a frequent occurrence in malignant diseases [1]. After treatment of the primary tumour and exclusion of other extrapulmonary manifestation, pulmonary metastasectomy is a well-established first-line treatment option in eligible patients [2–5]. In patients with significantly reduced lung function or other contraindications to surgery, a limited number of metastases can be treated by thermoablation [6] or stereotactic radiotherapy [7]. For patients with multiple, unresectable pulmonary metastases or multiorgan metastases, a systemic approach with chemotherapy and immunotherapy is warranted. However, particularly chemotherapy is associated with a considerable systemic toxicity. In cases with lung-dominant disease, an organ-based approach could potentially increase treatment efficacy and reduce systemic toxicity at the same time [8]. So far, treatment strategies with intensified isolated organ treatment only play a minor role in clinically established treatment concepts in those patients.

In whole lung irradiation, the reduced tolerance of the lungs to irradiation limits its usefulness in malignant diseases other than paediatric tumours and has been critically appraised in recent literature due to its toxicity and a non-superior survival [9–11]. Alternatively, chemotherapeutic in vivo lung perfusion in addition to metastasectomy has been proposed as an organ-based treatment approach by several authors. In terms of pulmonary concentrations of the cytostatic agent compared to intravenous injection, cytostatic activity, and pulmonary toxicity, the results have been promising [12–16]. Still, *in vivo* lung perfusion has to be considered an investigational technique that needs further evaluation in phase II and III trials before it can transition to clinical practice [8, 17]. Due to the invasiveness of this procedure including bilateral thoracotomies and cannulation of the pulmonary vessels, the use of this method in a non-curatively intended treatment concept is unlikely. Palliative patients with lung-dominant disease might benefit from a less invasive treatment.

Selective transpulmonary chemoembolisation (TPCE) using degradable starch microspheres (DSM) as a temporarily embolisation agent to increase the intratumoural dose of the chemotherapeutic agents has been already described in oncological patients with various cancers [18, 19]. However, DSM-TPCE of the whole lung might be a more promising concept, especially in patients with diffuse metastatic pulmonary disease. Until now, this organ-based approach by treating the whole lung has been mostly investigated in a rat model [20, 21]. A recent study by Barabasch et al. [22] thoroughly evaluated this approach in a pig model using doxorubicin and commercially available DSM as embolic agents (Embocept S^TM^, Pharmacept®, Berlin, Germany), underlining the feasibility of this technique. However, these data still have to be considered as preliminary and further evaluation is necessary to apply this promising technique in humans.

*Ex vivo* lung perfusion and surgical reimplantation of isolated lobes [23] are well-established techniques in lung transplantation. During *ex vivo* lung perfusion, physiologic conditions are mimicked by an extracorporeal membrane oxygenator circuit using a hyper-oncotic albumin-based perfusion solution. Lung ventilation is achieved by a ventilator and this made it possible to re-evaluate lung function of marginal donors and thus increase the number of lungs available for transplantation [24–26]. While the technique of *ex vivo* lung perfusion has been investigated before [27], recent developments made it possible to transfer this technique to surgically resected human lobes as a research platform, *i.e.*, to isolated lung perfusion (ILP) [27, 28].

The aim of this study was to use establish an ILP model to study transpulmonary embolisation (TPE) using DSM and to evaluate its impact of on human lung lobes, thus laying the foundation for further research and possible clinical applications of whole lung DSM-TPCE.

## Methods

### Patients

The study was approved by the institutional review board of the University Duisburg-Essen (date of approval: 10.01.2018, code of approval: 17-7802-BO). Patients gave written informed consent ahead of surgery. All patients had either a verified or suspected lung cancer in stage I/II according to the 8th edition of the Union for the International Cancer Control TNM classification of malignant tumours [29], underwent complete preoperative staging, and had surgical resection recommended after interdisciplinary tumour board discussion. The protocol was designed to ensure that routine histopathological examination that was not hampered by the experiments.

### Specimen preparation and isolated lung perfusion

Immediately after retrieval during surgery, the lobes were topically cooled with cold saline and flushed out both ante- and retrogradely with 1 L of cold (4 °C) buffered preservation solution (Perfadex Plus®, XVIVO, Göteborg, Sweden) with 5,000 IU of unfractionated heparin added. Silicone cannulas were sutured on the vascular and bronchial stumps with single running sutures (4/0 Prolene®, Johnson & Johnson Medical GmbH, Norderstedt, Germany). All procedures were performed by the same board certified thoracic surgeon with 5 years of experience. After adequate inflation with an Ambu-bag, the lungs were stored at 4 °C until ILP.

ILP was performed in the computed tomography (CT) room according to our institutional protocol [28]. Perfusion was achieved with a Cardiohelp pump (Maquet, Gettinge, Rastatt, Germany) and a modified extracorporeal membrane oxygenation circuit. The circuit was primed with 1220ml of hyper-oncotic acellular colloidal perfusate (32.8 g/L succinated gelatine; 32.8 g/L human albumin; 6.6 g/L glucose). After initial warming-up, lungs were ventilated (Dräger, Evita XL, Lübeck, Germany) in a protective manner according to the calculated tidal volume of the patient and the number of perfused lung segments (6ml/kg). Ventilation parameters were as follows: 8 bpm, ½ inspiratory/expiratory ratio, 0.4 fraction of inspired oxygen, and 5–10 cm H_2_0 of positive end-expiratory pressure. Perfusion flow was maintained at 40% of the estimated cardiac output normalised on the number of perfused lung segments. Dynamic lung compliance was calculated according to following formula: Tidal volume/(peak airway pressure/positive end-expiratory pressure).

### Transarterial lung embolisation

**Fig. 1:**
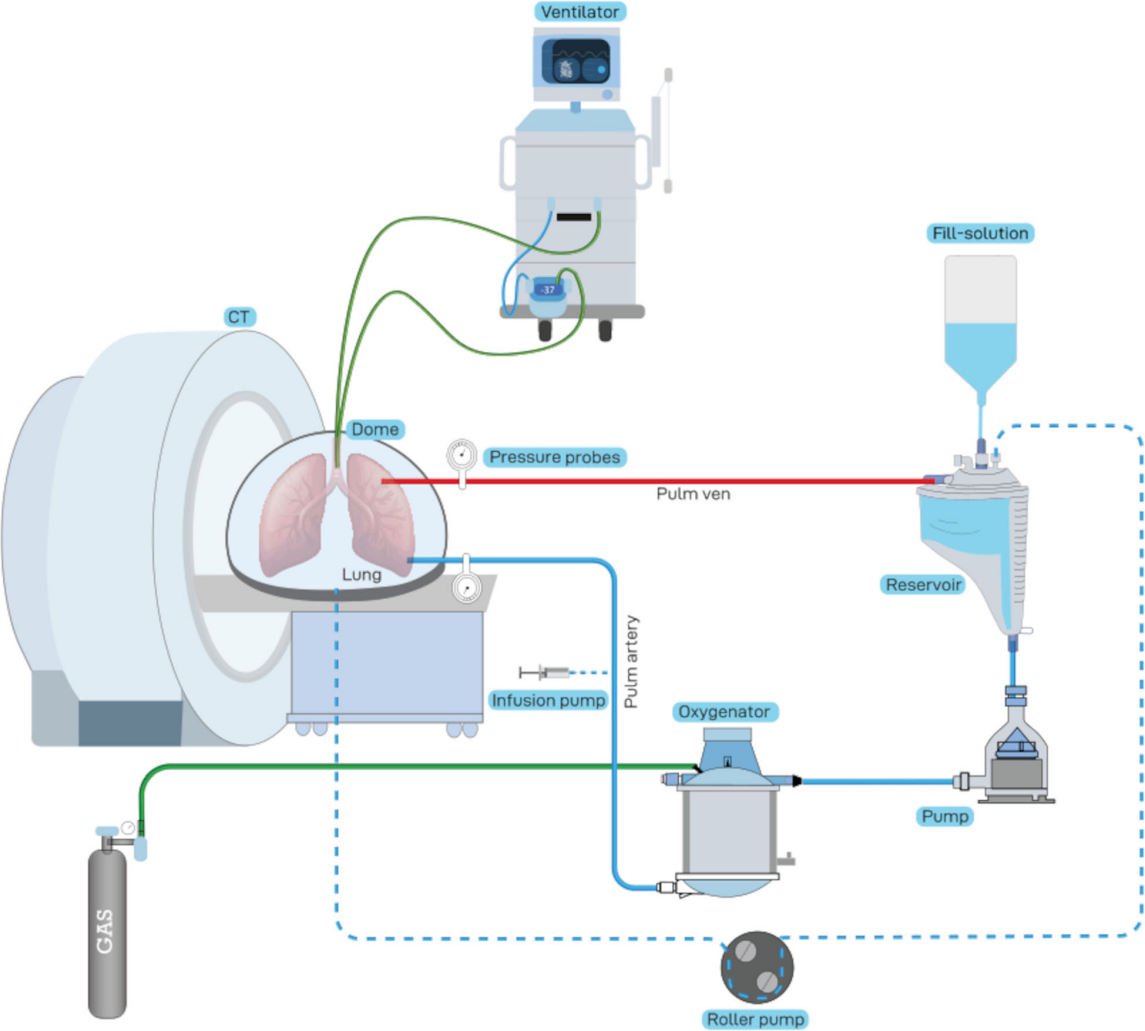
Schematic diagram of the experimental setting. The lung lobe (represented by a double lung for ease of understanding) is placed in a plexiglass dome in the computed tomography (CT) gantry. Green lines mark ventilation/gas tubes. The dashed line corresponds to the venous return of leaked perfusate. Degradable starch microspheres and contrast agents are added to the pulmonary artery. The extracorporeal membrane oxygenator is placed in front of the CT gantry and the ventilator is placed behind the CT gantry. The contrast agent injector, a monitor for pulmonary artery pressure measurements and the automated syringe infusion pump are also visible

**Fig. 2:**
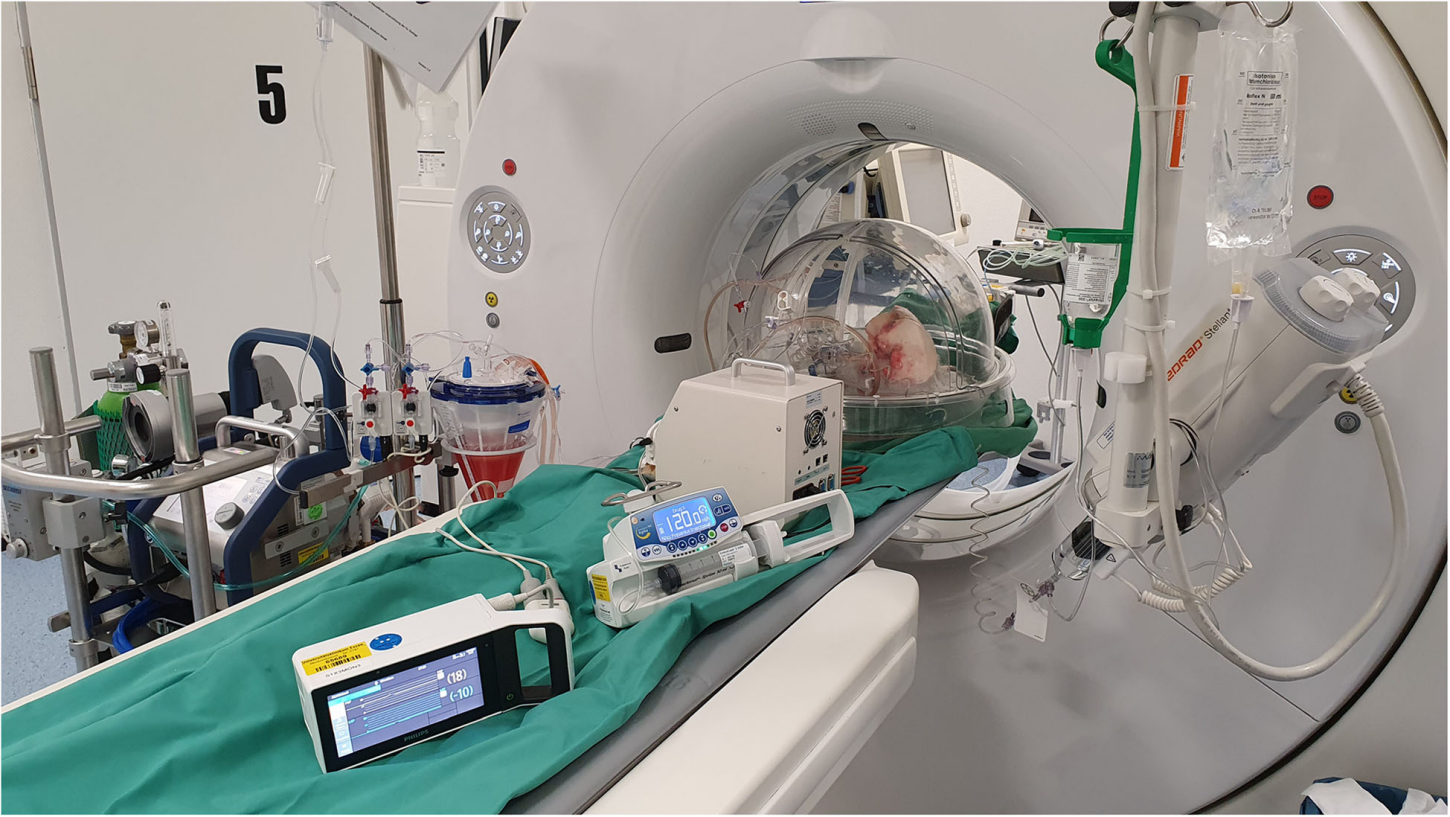
Actual setup. The lower lung lobe is positioned in the computed tomography gantry

Before TPE, the explanted lung lobe was positioned in the CT gantry of a Somatom Definition AS (Siemens Healthineers, Forchheim, Germany) for repeated CT scans and CT perfusion imaging (CTPI) during the experiment (Figs. 1 and 2).

CT scans of the whole lobe were acquired under continuous ventilation with a slice thickness of 1mm and a pitch of 0.6. To optimise image quality, CareKV^TM^ (preset 120kV) and CareDose^TM^ (preset 77mAs) were used. For CTPI, CT data were acquired continuously for 60 s with a time increment of 1 s and fixed CT acquisition parameters (100 kVp, 150 mAs, 10 mm slice thickness). Contrast agent (Ultravist® 300, Bayer Vital GmbH, Leverkusen, Germany) was administered using an automated contrast agent injector. Ten seconds after the start of continuous CT acquisition, a total of 15 mL contrast agent was injected followed a 15 mL saline flush with an injection rate of 2 mL/min using an automated syringe infusion pump (Injectomat® MC Agila, Fresenius Kabi, Bad Homburg vor der Höhe, Germany)

**Fig. 3:**
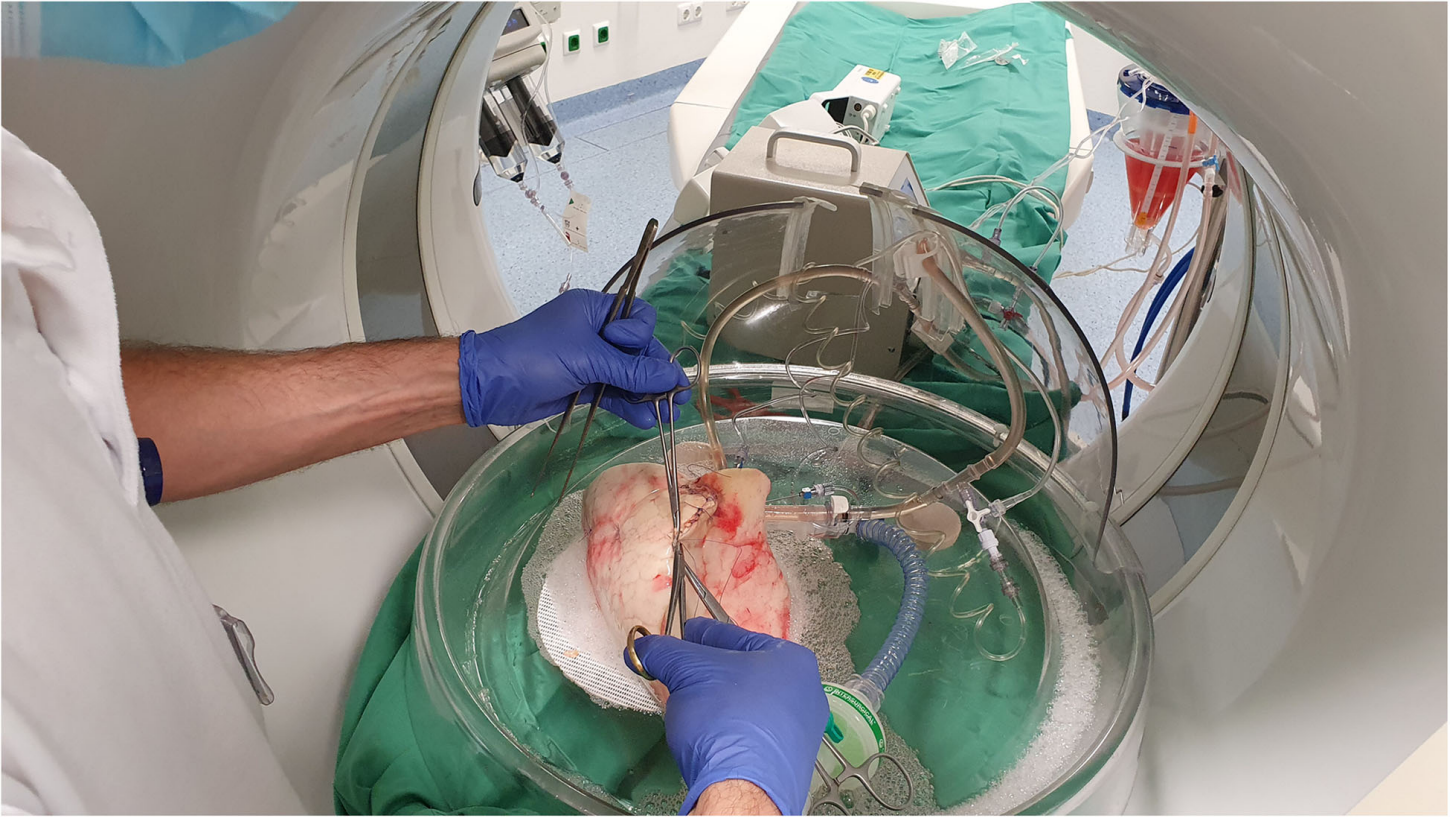
Surgical biopsy taken from the periphery of the lung lobe for microscopy analysis

**Fig. 4:**
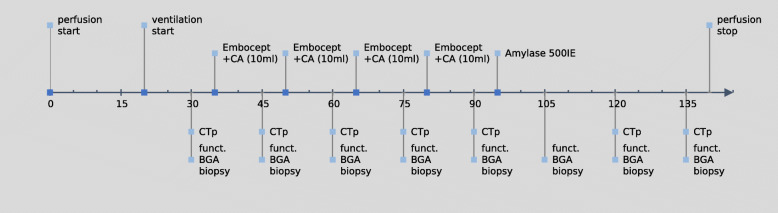
Timeline of the experiment. *BGA* Blood gas analysis, *CA* Contrast agent, *CTPI* Computed tomography perfusion imaging, *Funct.* Functional assessment. *x* axis values given in min

Lung perfusion was started at t0, and both flow and temperature were continuously increased until normothermia (37 °C) was reached. Ventilation and deoxygenation of the perfusate was started simultaneously. After and an initial CT scan (t30) of the lobe, baseline CTPI was performed. Then, (at t35) TPE was initiated with 10 mL of a mixture of 12.5 mL of contrast agent and 450 mg of DSM with a sphere size of 50 ± 7 μm in a solution of 7.5 mL (Embocept^TM^ S, PharmaCept GmbH, Berlin, Germany) at an injection rate of 2 mL/min via the arterial limb. This procedure was repeated at prespecified timepoints (t45, t60, and t75). After the assessment at t90, 500 units of alpha-amylase (Merck KGaA, Darmstadt, Germany) were injected at 2 mL/min into the arterial limb (diluted in 50 mL of perfusion solution) to hydrolyse the DSM. After complete administration of the amylase, two additional CT scans and CTPI were performed. Surgical biopsies of 1.0 to 2.5 cm in size were taken from the periphery of the lobe (Fig. 3), and functional assessments, *i.e*., pulmonary artery pressure (PAP), airway pressure, pulmonary vascular resistance (PVR), and a blood gas analysis were performed throughout the experiments according to the study timeline (Fig. 4). The perfusion was stopped after the last assessment.

### Histopathological analysis

Histopathological specimens were stored directly after sampling in 4% neutral buffered formalin. For microscopy analysis, complete biopsy specimens were embedded and serial sections of 3 to 4 μm thickness were stained with haematoxylin and eosin. A pathologist, blinded to the timepoints of histopathological sampling, analysed all slides. At microscopy analysis, starch particles appeared as pale bluish spherical structures within blood vessels without any inflammatory reaction. Single DSM particles had a size of 20 to 30 μm and tended to aggregate with a cluster size > 150 μm. Affected small lung vessels had a size of 50 to 300 μm. Due to differences in lung parenchyma including emphysematous or atelectatic changes, an area-based quantitative analysis was considered as unfavourable. Therefore, a semiquantitative ordinal score was used to describe the extent of embolisation (0 = no particles; 1 = single particles, no aggregates; 2 = single particles, few aggregates (< 50% of all starch particles consisting of aggregates); 3 = ≥ 50% of starch particles appeared as large aggregates).

At the end of the procedure, the lobe resection specimens were processed for routine histopathological work-up.

### CTPI analysis

Images of CTPI were analysed using the Syngo.CT Dynamic Angio workflow of the syngo.via software (VB40, Siemens Healthineers, Forchheim, Germany). In the upper, mid, and lower periphery of the lung lobe, three non-overlapping regions of interests (ROIs) were drawn with a size of 3 to 5 cm^2^ depending on the size of the lobe and a central ROI in the pulmonary artery for every CTPI acquired at t30, t45, t60, t75, t90, t105, and t120 for each patient to determine time to peak (TTP). To correct for differences caused by the length of the vascular anastomoses as well as artifacts caused by lung consolidation, TTP of ROI in the pulmonary artery was subtracted from the mean TTP value of the three ROI obtained in the upper, mid, and lower periphery of the lung lobe for each patient and each timepoint.

### Statistical analysis

Data are presented as mean ± standard deviation or median and range (minimum and maximum) according to the normal/near-normal or non-normal distribution. Data plotting and analysis were performed with Graphpad Prism 9.0 (GraphPad Software, San Diego, CA, USA). Linear regression analysis and one-way ANOVA were used to evaluate parameter dynamics throughout experiments. The 95% confidence intervals where computed and plotted on graphs. *p* values < 0.05 were considered as statistically significant. Due to the explorative nature of this study, no correction for multiple testing was performed.

## Results

### Cases

All patients either had a verified (*n* = 5) or suspected (*n* = 1) non-small-cell lung cancer in stage I/II as underlying diagnosis. Median age was 66 years (range 61−71). History of nicotine abuse was 32.5 pack-years (range 20−50). Median forced expiratory volume in 1 s was 80.5% of predicted (range 57−89%) while diffusion capacity of the lungs for carbon monoxide was 68% of predicted (range 26−102%). Patients underwent lobectomy (*n* = 5) or bilobectomy (*n* = 1), either via thoracotomy (*n* = 5) or via video-assisted thoracoscopic surgery (*n* = 1). Median warm ischemic time of the resected lobes was 30 min (range 12−60) followed by a median cold ischemic time of 261 min (range 159−347). Patient and ILP data are summarised in Table 1.

**Table 1:** Patient demography and oncological data of the investigated patients

Case	1	2	3	4	5	6
Age (years)	63	67	71	61	66	66
Sex	Male	Male	Female	Female	Female	Female
Emphysema	No	Yes	No	No	No	No
Smoker (packs/year)	Ex (35)	Yes (35)	Ex (20)	Ex (30)	Yes (28)	Yes (50)
FEV1 (%)	81	57	72	89	80	81
rTLC (%)	97	115	107	102	103	98
DLCO (%)	74,5	25,9	72,3	53,5	102,2	63,7
Surgical access	Thoracotomy	Thoracotomy	uVATS	Thoracotomy	Thoracotomy	Thoracotomy
Lobe	Right LL	Right LL	Right LL	Right LL	Right ML+LL	Right LL
Perfused segments	5	5	5	4 ^a^	7	5
Histopathology	SCC	SCC	SCC	AC	SCC	SCC ^b^
TNM staging and grading ^c^	pT1b pN0 G3 L0 V0 R0	pT2a pN1 G3 L1 V0 R0	pT2a pN0 G3 L0 V1 R0	pT1b pN0 G2 L0 V0 R0	pT1c pN1a G2 L0 V1 R0	ypT0 ypN0 GX L0 V0 R0
Warm ischemia time (min)	40	60	18	46	20	12
Cold ischemia time (min)	310	347	194	159	230	292
Total ischemia time (min)	350	407	212	205	250	304

*AC* Adeno carcinoma, *DLCO* Diffusion capacity of the lungs for carbon monoxide, *Ex* Ex-smoker, *FEV1* Forced expiratory volume in one second, *LL* Lower lobe, *PY* Pack/year, *rTLC* Real total lung capacity, *SCC* Squamous cell carcinoma, *uVATS* Uniportal video-assisted thoracoscopic surgery

^a^Patient 4 underwent a wedge resection before lobectomy; thus, the perfused segments were estimated at 4

^b^Patient 6 had a neoadjuvant treatment with cisplatin/vinorelbine and 45 Gy of irradiation; initial tumour stage was cT2 cN2 cM0

^c^According to the 8th UICC TNM edition (https://www.uicc.org/resources/tnm/publications-resources)

**Fig. 5:**
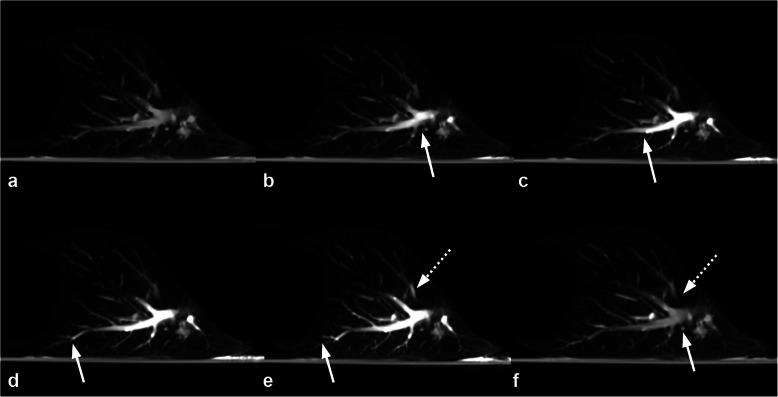
Computed tomography perfusion imaging of case #3 at t30. While unenhanced pulmonary vessels can be observed prior to contrast media injection (**a**), a gradual filling of the arterial vessels can be observed (white arrow, from **b** to **e**), followed by a filling of the pulmonary veins (dotted arrow in **e**), and a complete washout of the contrast agent

**Fig. 6:**
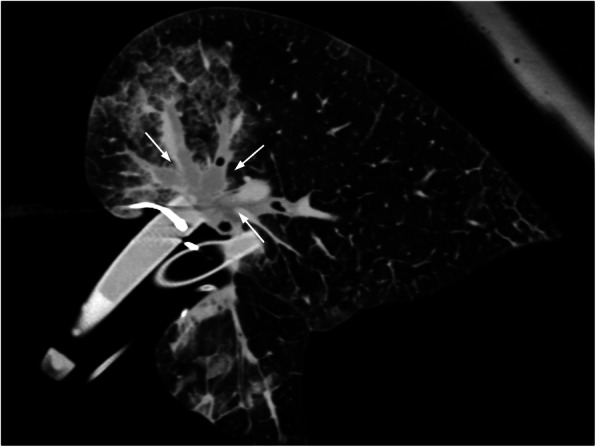
Extensive central pulmonary embolism (white arrows) of the main pulmonary arteries and all segmental branches (case #5)

**Fig. 7:**
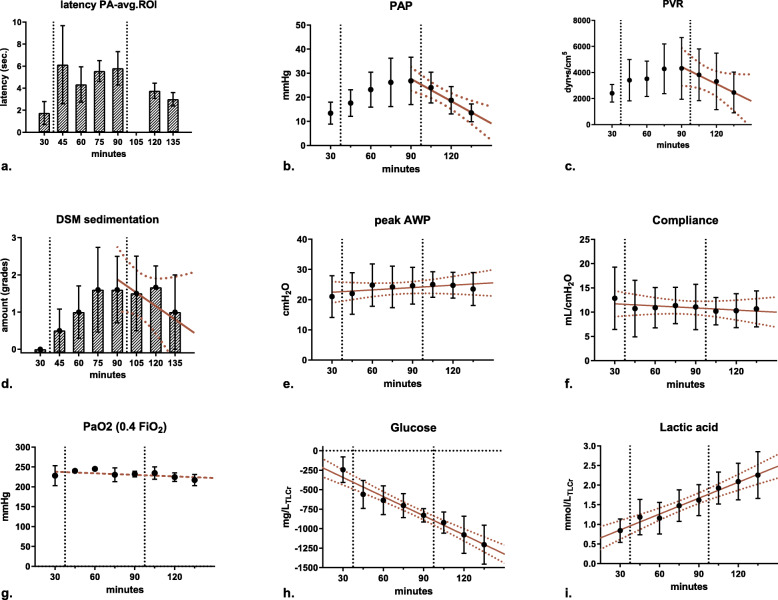
Recorded parameters during isolated lung perfusion. The first dashed vertical line in each graph indicates the first transpulmonary embolisation. The second vertical line indicates the administration of 500 units of alpha-amylase. Red solid lines indicate the linear regression line and red dashed lines represent the confidence band. *avg.ROI* Average to regions of interest, *AWP* Airway pressure, *DSM* Degradable starch microspheres, *PA* Pulmonary artery, *PaO*_*2*_ Partial pressure oxygen, *PAP* Pulmonary arterial pressure, *PVR* Pulmonary vascular resistance. *x* axis values given in min

Five out of six experiments were carried out successfully. In one case (#5), ILP was aborted as the pulmonary arteries were fully obstructed by blood clots extending into the periphery of the vasculature and not amenable to removal. This was identified at the second CT (at t45, Fig. 5) and led to a very high pulmonary arterial pressure (> 40 mmHg) and a fulminant lung oedema before any DSM could be administered (Fig. 6). This experiment was omitted from analysis. The remaining cases were included and recorded parameters are presented in Fig. 7

### Vascular parameters

During the administration of DSM (from t35 to t90), both PAP and PVR increased significantly. Mean PAP increased by 200% from 13.4 ± 4.6 to 26.8 ± 9.9 mm Hg (PAP slope 95% confidence interval [CI] 0.104 to 0.483, *p* = 0.0044 ) and PVR increased by 180% from 2401 ± 684 to 4314 ± 237 dyn·s·cm^−5^ (PVR slope 95% CI -0.65 to 77.82, *p* = 0.053). Accordingly, TTP measured in CTP increased significantly to a plateau of approximately 6 s from the main pulmonary artery to the defined average ROI (1.7 ± 1 s at t30 *versus* 6.1 ± 3.5 s at t45, *p* = 0.046). At t90, a total of 900 mg DSM had been applied to the lower lung lobes. This increase in PAP and PVR led to a significant leakage at the arterial cannulation site in two cases. After the addition of alpha-amylase at t95, the effect was reversed. Cannulation leakage ceased, and PAP, PVR, and TTP reverted to values comparable to baseline: at t135, PAP was 13.5 ± 3.7 mm Hg, PVR 2465 ± 1,561 dyn·s·cm^−5^, and TTP 3.0 ± 0.6 s.

### Airway parameters and gas exchange

Throughout embolisation and subsequent DSM hydrolysis, airway pressure, lung compliance, and oxygenation remained steady and a linear regression analysis failed to show any significant slope deviation from zero. Airway pressure was 21 ± 7 cm H_2_O at t30, 24.6 ± 6 at t90, and 23.5 ± 5 at t135; slope 95% CI -0.03 to 0.08; *p* = 0.367. The lung compliance was 12.8 ± 6 mL/cm H_2_O at t30, 11.1 ± 5 at t90, and 10.7 ± 4 at t135; slope 95% CI -0.057 to 0.028; *p* = 0.496. pO_2_ was 228 ± 25 mm Hg at t30, 232 ± 7 at t90, and 217 ± 14 at t135; slope 95% CI -0.28 to 0.017; *p* = 0.080.

### Metabolism

During ILP, glucose metabolism and lactic acid production was measured repeatedly. Normalised (on total lung capacity in litres) cumulative glucose consumption was 829 ± 86 mg/L at t90 and 1,206 ±250 mg/L at t135. Lactic acid accumulation, normalised on total lung capacity (TLC), reached 2.3 ± 0.6 mmol/L at t135. No significant deviations from the fitted regression line were observed. Average glucose consumption over time (-8.19 mg/min/L TLC, 95% CI -9.7 to -6.66, *R*^2^ = 0.97) and lactic acid buildup (0.014 mmol/min/L TLC, 95% CI 0.010 to 0.017; *R*^2^ = 0.58) were higher than in previous reports [27].

### Histopathology

**Fig. 8:**
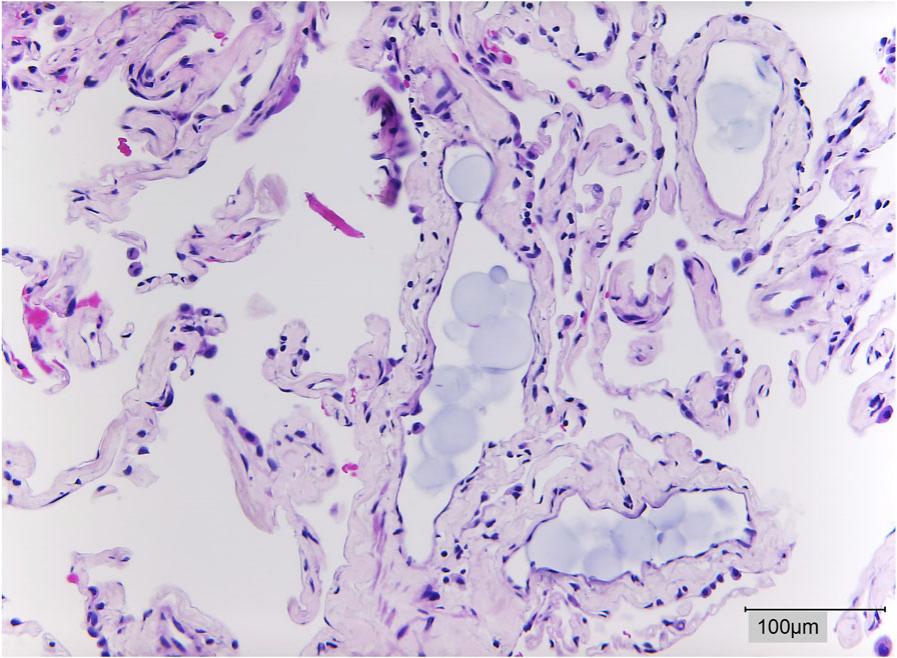
Histological section of sedimented DSM a t90 with biggest aggregates of 150 μm, sample was graded as 3 according to the semiquantitative ordinal score

Analysis of specimens was performed at each timepoint. With repeated administration of DSM, embolisation in the capillaries increased in HE staining. Starch hydrolysis was not observed before amylase was added to the perfusate at t95. Embolisation grades (0 to 3) increased significantly until t90 (slope 95% CI 0.027 to 0.066, *p* < 0.0001) and decreased after addition of alpha-amylase (slope 95% CI -0.060 to 0.012, *p* = 0.165). A representative example of DSM embolisation at t90 is provided in Fig. 8.

## Discussion

Whole lung DSM-TPCE has the potential to supplement palliative treatment of pulmonary metastases in various oncological diseases, particularly those in which lung metastases representing the leading cause of death. However, further basic research is necessary to understand the histological and physiological changes induced by this promising technique prior to clinical studies. In this study, we could successfully establish a reliable human *ex vivo* ILP model to analyse the physiological and histopathological effects of whole lung DSM-TPE on a living organ including the dissolution of DSM using alpha-amylase.

Furthermore, this pilot study yielded the following main results. First, repeated histological sampling revealed the intended continuous increasing obstructive effect of DSM-TPE on the lung vasculature. Second, the histological changes of whole lung DSM-TPE as well as their reversal after alpha-amylase injection could be successfully monitored using arterial pressure measurements and repeated CTP imaging. Third, the application of a maximum of 900-mg DSM into a lower lung lobe led to a drastic increase of the intra-arterial pressure and anastomotic leakage and thus should not be exceeded.

Despite general advances in oncology, the treatment of advanced pulmonary metastatic disease still relies on systemic chemotherapy. To confine the chemotherapeutic effect to the lung and increase the cytotoxic effect on cancer cells, *in vivo* ILP might be an important option. However, most experience is derived from studies performed on various animal models and a selected number of phase I trials [8, 16, 17, 30, 31]. A phase II study recently published by Den Hengsten et al. [32]. indicates the potential role of *in vivo* ILP as an adjunct to metastasectomy in patients with limited pulmonary disease to decrease cancer recurrence rate. However, the introduction of this technique as a possible treatment option in a non-curative treatment regimen or in patients that have already undergone previous pulmonary metastasectomies has to be doubted. Here, a minimal invasive, catheter-based treatment approach to perform a whole lung DSM-TPCE might be a useful treatment concept. While first clinical results on selective TPCE in pulmonary metastases have been published by Vogl et al. [18, 19], to the best of our knowledge, clinical studies focusing on whole lung DSM-TPCE have not been published until now.

Recent studies evaluated the effects of whole lung DSM-TPCE in small and large animal models [21, 22, 33]. Pohlen et al. [21, 33] analysed the benefits of whole lung DSM-TPCE in a rat and in a pig model. The authors could observe a markedly increased concentration of the chemotherapeutic agent, carboplatin, in the tumour in comparison to other types of administration such as intravenous injection or ILP. Here, the authors could observe a markedly increased concentration of the chemotherapeutic agent, carboplatin, in the tumour in comparison to other types of administration such as intravenous injection or ILP [33]. Furthermore, the feasibility of this approach could be transferred to a large animal model and the physiological effects of whole lung DSM-TPCE could be demonstrated. Barabasch et al. [22] established a pig model and evaluated whole lung DSM-TPCE with doxorubicin. In a pig model, both Barabasch and Pohlen observed temporary physiological changes during DSM-TPCE, but no lasting lung damages was observed in the histological evaluation [21, 22].

Although examining DSM-TPCE in a living organism has the advantage to observe physiological adaption processes during the injection of DSM as well as late effects of this procedure, animal models have their shortcomings. Repeated intraindividual histological sampling during the embolisation is not possible in a small animal model due to the small organ size and is associated with relevant technical problems in a large animal model. Furthermore, differences between the different animal models and human lungs impede a direct knowledge transfer to clinical studies, especially concerning necessary doses of DSM or chemotherapeutic agents. Therefore, a different approach is necessary to elucidate the immediate effects caused by whole lung DSM-TPCE and close the gaps between experience gained in animal models and clinical applications.

Our study proves that it is possible to reproduce the observations found in animal models in a human *ex vivo* ILP model. While changes in pulmonary arterial pressure were comparable to the results observed in the study by Pohlen et al. [21], we could prove the continuously increasing microvascular occlusion caused by DSM clusters histologically, leading to a delayed peripheral lung parenchyma perfusion observed by CTP. These changes could be reversed by the injection of alpha-amylase. Furthermore, gas exchange and mechanical properties of the airways (measured by pO_2_, airway pressure. and lung compliance) remained unaltered throughout the experiments. This corroborates that DSM-TPE does not lead to a relevant capillary leakage and thereby to extravascular fluid shift into the interstitial space or the alveoli, which would impede normal lung function by restricting the physiological gas exchange due to pulmonary oedema. Thus, our *ex vivo* ILP model might be an excellent opportunity to understand the short-term effects of whole lung DSM-TPE in the human lung. Further studies should evaluate the short-term impact of different chemotherapeutic agents in DSM-TPCE protocols on human lung parenchyma. Therefore, questions raised by published animal models could be answered by further studies on *ex vivo* ILP models closing important gaps of knowledge in our understanding of DSM-TPCE.

Our study has some limitations. First, the sample size in our study is small and no correction for multiple testing was performed. However, due to the novelty of our approach, we aimed to prove the feasibility of a human *ex vivo* ILP model for further evaluation of whole lung DSM-TPCE. Furthermore, our results are sufficient to demonstrate the feasibility of our human *ex vivo* ILP model and provide insights into the histological and physiological changes induced to nonmetastatic pulmonary tissue by whole lung DSM-TPE.

However, its effects on tumour tissue have to be investigated further. Second, alpha-amylase was only administered at the end of the experiment via the arterial limb, while the addition of low-dose alpha-amylase to the perfusate would have provided the opportunity to observe continuous DSM dissolution. This study design was chosen deliberately to allow for better investigation of potentially deleterious effects of DSM-TPE on the physiological lung function. Third, possible effects of DSM-TPCE caused by the combined emboligenic and chemotherapeutical effect of this procedure cannot be derived from our results. Therefore, further studies analysing the effects of different chemotherapeutic agents in DSM-TPCE in larger samples are mandatory to understand this new procedure thoroughly. Fourth, some physiological changes observed in animal models by other groups such as the heart rate cannot be evaluated in our model. Additionally, long-term changes of DSM-TPE/TPCE cannot be investigated in our model as physiological conditions in *ex vivo* ILP can only be sustained for few hours [28]. However, *ex vivo* ILP offers the possibility to perform arterial pressure measurements as well as CTPI and histological sampling at multiple timepoints during the procedure and to perform intraindividual and not only interindividual comparisons as in some small animal models. Furthermore, a distinct advantage of our *ex vivo* ILP model in comparison to previously used small and large animal models is the opportunity to analyse the effects of DSM-TPE on a human and not an animal lung.

In conclusion, we showed that a human *ex vivo* ILP model is a possible option to continuously observe and monitor the effects of whole lung DSM-TPE and thus could be a tool to assess the effects of whole lung DSM-TPCE on a human organ.

## Data Availability

The datasets generated and/or analysed during the current study are not publicly available due to continuous research on this topic but are available from the corresponding author on reasonable request.

## Abbreviations

CI: Confidence interval
CT: Computed tomography
CTPI: CT perfusion imaging
DSM: Degradable starch microspheres
ILP: Isolated lung perfusion
PAP: Pulmonary arterial pressure
PVR: Pulmonary vascular resistance
ROI: Region of interest
TPCE: Transpulmonary chemoembolisation
TPE: Transpulmonary embolisation
TTP: Time to peak
